# The Role of Enhanced Cognition to Counteract Detrimental Effects of Prolonged Bed Rest: Current Evidence and Perspectives

**DOI:** 10.3389/fphys.2018.01864

**Published:** 2019-01-23

**Authors:** Uros Marusic, Voyko Kavcic, Rado Pisot, Nandu Goswami

**Affiliations:** ^1^Institute for Kinesiology Research, Science and Research Centre Koper, Koper, Slovenia; ^2^Department of Health Sciences, Alma Mater Europaea – European Center Maribor, Maribor, Slovenia; ^3^Institute of Gerontology, Wayne State University, Detroit, MI, United States; ^4^Head of Research Unit: “Gravitational Physiology, Aging and Medicine”, Otto Loewi Research Center of Vascular Biology, Immunity and Inflammation, Medical University of Graz, Graz, Austria

**Keywords:** non-pharmacological countermeasures, aging, cognitive training, geriatrics, falls prevention

## Abstract

Prolonged periods of physical inactivity or bed rest can lead to a significant decline of functional and cognitive functions. Different kinds of countermeasures (e.g., centrifugation, nutritional, and aerobic interventions) have been developed to attempt to mitigate negative effects related to bed rest confinement. The aim of this report is to provide an overview of the current evidence related to the effectiveness of computerized cognitive training (CCT) intervention during a period of complete physical inactivity in older adults. CCT, using a virtual maze navigation task, appears to be effective and has long-lasting benefits (up to 1.5 years after the study). Moreover, enhanced cognition (executive control) reduces decline in the ability to perform complex motor-cognitive dual-tasks after prolonged period of bed rest. It has been demonstrated that CCT administration in older adults also prevents bed rest stress-related physiological changes [these groups showed minimal changes in vascular function and an unchanged level of brain-derived neurotrophic factor (BDNF)] while control subjects showed decreased peripheral vascularization and increased plasma level of the neurotrophin BDNF during a 14-day bed rest. In addition, the effects of CCT are evident also from the brain electrocortical findings: CCT group revealed a decreased power in lower delta and theta bands while significant increases in the same EEG spectral bands power were found in control subjects. If we consider an increase of power in delta band as a marker of cortical aging, then the lack of shift of EEG power to lower band indicates a preventive role of CCT on the cortical level during physiological deconditioning induced by 2-week bed rest immobilization. However, replication on a larger sample is required to confirm the observed findings. Applications derived from these findings could be appropriate for implementation of hospital treatment for bed ridden patients as well as for fall prevention programs.

## Introduction

Hospitalization and prolonged bed rest represent major risk factors for older persons, often resulting in irreversible deterioration in functional status and a significant decline in the quality of life (Blain et al., 2016; Bousquet et al., 2017; Goswami, 2017; Goswami et al., 2017). The proportion of older persons (defined as ≥65 years) is increasing worldwide, and is projected to double by the year 2030 with an expansion in life span of another 10 years by the year 2050 (United Nations, 2013). In addition, the number of super-olds (≥80 years) is expected to triple, from 22 to 61 million from year 2008 to 2060, respectively (European Commission, 2009). It has been shown that the greater the age, the stronger the association of bed confinement with functional loss and failure to recover during hospitalization (Covinsky et al., 2003) or in the nursing home setting (Fortinsky et al., 1999). As a consequence of this trend in population aging, higher demands for public health and aging services together with concomitant increases in health care costs are foreseen (Blain et al., 2016; Bousquet et al., 2017; Goswami, 2017).

The bed rest model was first introduced in the 1960s to simulate acute adaptations to the microgravity environment involved in space flights (Adams et al., 2003; Goswami et al., 2015a). Indeed, bed rest confinement, especially during prolonged hospitalization, could be modeled on the so-called bed rest protocol where healthy participants spend a number of days in a horizontal or (more extreme) head-down tilt bed rest condition. The negative adaptations of the cardiovascular system were observed to be similar in spaceflight and bed rest confined persons (Goswami, 2017). The negative effects of bed rest confinement, however, occur ten times faster than those that arise due to the normal aging process (Vernikos and Schneider, 2009). Previous research has mainly focused on the effects of prolonged bed rest on cardiovascular and musculoskeletal physiology (Pavy-Le Traon et al., 2007; Pisot et al., 2016; Gao et al., 2018). With improved technological development and increasing availability of brain imaging techniques, changes and adaptations of the central nervous system can be more effectively studied and the application of these techniques has become of greater interest in bed rest research (Koppelmans et al., 2017; Van Ombergen et al., 2017a,b; Gandarillas and Goswami, 2018). Typically, past bed rest studies included young and healthy participants and were designed to simulate the microgravitational environment and its accompanying effects, relevant for space flight missions (Blaber et al., 2013; Goswami et al., 2013; Cvirn et al., 2015; O’shea et al., 2015; Waha et al., 2015). Both horizontal and head-down tilt bed rest protocols have been implemented. Important differences in the two protocols such as in the adaptation in tissue fluid redistribution and hydrostatic pressures, however, need to be taken into account (Hargens and Vico, 2016).

The aim of this review is to summarize the current scientific evidence regarding bed rest-related impacts on mainly cognitive outcomes of healthy adults, and to highlight the importance of non-physical countermeasures such as cognitive training. The primary application of such scientific knowledge is therefore to provide the basis for the development of possible countermeasures both for the negative consequences of bed rest confinement and space flight microgravity, in particular in regard to negative bed rest effects in older persons during hospitalization. Such countermeasures are potentially extendable to addressing important consequences of the aging process in general. Emphasis is placed in this review on cognitive countermeasures to prevent not only cognitive but also sensorimotor adaptations that occur during acute and chronic situations involving hospitalization, prolonged physical inactivity, and the general aging process.

## Search Strategy and Study Selection

Scientific literature in English language was acquired through searches conducted on PubMed/MEDLINE (NLM), Embase, and Web of Science databases until October 1, 2018. A search for manuscripts on “cognitive training” and “bed rest” (with specific deviations of keyword combinations, such as “hospitalization,” “cognitive intervention,” “brain training,” “mental training,” etc.) was conducted and yielded 807 results. Furthermore, reference sections of included manuscripts were inspected to identify additional manuscripts of interest. Our results showed that there were no other studies besides “Bed Rest Study – PANGeA, Valdoltra 2012 (Slovenia, EU),” which investigated effectiveness of cognitive interventions during bed rest. Therefore, the final search consisted of four manuscripts (Goswami et al., 2015b; Marusic et al., 2015, 2018a; Passaro et al., 2017) that represent the same bed rest campaign, while each manuscript reported results based on different outcome measures.

## Impact of Reduced Physical Activity and Bed Rest on Functional and Cognitive Outcomes

The positive role of physical activity in maintaining effective mobility is well-established by research and promoted by the popular media (Hui and Rubenstein, 2006; Muller et al., 2016). Reduction of the appearance of illness and chronic disease, improvements in gait and balance, as well as reduction in the risk of falls are among the most important advantages of engagement in physical activity by elderly people (Kovacs et al., 2013). A recent review highlighted the fact that up to 82% of total brain gray matter volume can be modified by engaging in physical activity (Batouli and Saba, 2017). Also, several types of cognitive-motor interventions have been previously applied in an older adult population showing positive improvements in functional and cognitive performance (van Iersel et al., 2007; Fraser et al., 2017), which is recently supported by brain structural adaptations also in subjects with mild cognitive impairment (Maffei et al., 2017). However, seniors often have limited access and fewer opportunities to engage in physical exercise programs with a 50% dropout rate in such activities in the first 3–6 months (Allen and Morey, 2010). Regardless of the issues of limited access and/or lack of motivation for engaging in physical exercises, older adults are often forced to limit or even completely eliminate physical activity due to injuries or surgeries, which results in a specific syndrome referred to as the “disuse syndrome” (Bortz, 1984). Among the main characteristics of this syndrome are premature aging, skeletomuscular fragility, obesity, cardiovascular vulnerability and depression (Bortz, 1984).

In general, frail seniors are often limited by a disability- or a disease-related burden preventing their physical activity, and for them engaging in real-world situations involving complex locomotion where higher cognitive-resource demands are required can be a challenge. Reduced levels of physical activity have also been shown to be associated with increased risk of cognitive impairment and various types of dementia (Laurin et al., 2001). Interestingly, the results of bed rest studies do not always point toward the same conclusions. Over the years, various stressors that could lead to cognitive impairment after periods of hospitalization have been proposed. The increased levels of stress created by hospitalization itself with accompanying alternations of stress-response hormones and neuro-chemicals, as well as delirium, medications and polypharmacy, and depression are important factors in this regard (for a detailed review, see Mathews et al., 2014).

The majority of bed rest trials in which cognitive performance was assessed were carried out in a head-down tilt position and yielded contradictory results. For example, impaired cognition was found in a study of Lipnicki et al. (2009). These authors reported alterations of cognitive processes associated with decision making after 50 days of head-down tilt bed rest. After a similar length (45 days) of head-down tilt bed rest, Liu et al. (2012) observed worsening of executive functions. On the other hand, a 16-day head-down bed rest study did not report any changes in executive functions (Ishizaki et al., 2009). Moreover, in other studies, neither 17 days, nor 60 or 90 days of head-down tilt bed rest affected general cognitive functioning (Shehab et al., 1998; Seaton et al., 2009).

There is a scarcity of literature available reflecting cognitive outcomes after horizontal bed rest. After 14-day horizontal bed rest with young adults, (Dolenc and Petri, 2013) observed a minor improvement in mental visualization and no change in other assessed cognitive functions. In the same study, older individuals showed significant impairments in delayed recall (Dolenc and Petri, 2013), which was, however, not the case for those subjects who had cognitively stimulating environment during bed rest (Marusic et al., 2018a).

As extensively summarized in the “bed rest and cognitive functioning review” by Lipnicki and Gunga (2009), results from experimental studies with healthy young and older individuals are also not conclusive and do not unequivocally point in the same direction. More specifically, only eight of 17 bed rest studies included in that review reported significant detrimental effects on cognitive performance. Six studies reported unchanged cognitive functioning after bed rest, whereas three studies surprisingly showed improvements in cognitive performance. In the latter case, task exposure and practice effects could mask the underlying detrimental effect of bed rest on cognitive functioning (Lipnicki and Gunga, 2009), suggesting that eliminating practice effects in neuropsychological tests is important for the better evaluation of bed rest on cognitive functioning.

The majority of bed rest trials assessing cognitive performance before and after bed rest fail to address the underlying adaptation of the brain and subsequent correlational analysis between behavioral and neural outcome measures. Differences also exist among bed rest designs which vary in terms of amount of days that the participants were bedridden, type of the bed rest protocol used (e.g., horizontal or head-down tilt), as well as the motivation behind the studies. Some of these studies aimed at replicating spaceflight conditions, lack of sensory-motor stimulation and immobilization, and/or post bed rest recovery (Lipnicki and Gunga, 2009; Marusic et al., 2014b). Thus, an open question remain how CCT could be used as a general approach for improving cognitive performance in bed rest confined older subjects.

## Cognitive Training as a Possible Countermeasure During Prolonged Bed Rest

Cognitive training aimed at optimizing cognitive functioning and/or slowing brain aging has been extensively used, especially with healthy older adults. It generally involves guided practice on tasks representing different domains of cognition in order to increase or maintain particular cognitive functions such as memory or attention. Cognitive training programs are commonly run as a time-limited, daily sessions for a specified period of intervention (e.g., 1 hour per day for 5 days a week for a total of 20 sessions). The training tasks are often designed to present an increasing challenge to cognitive abilities and thereby induce learning. A variety of tasks and approaches have been used for cognitive training (for a detailed review, see Tardif and Simard, 2011), with most of the reviewed studies reporting significant improvements in cognitive functions associated directly to those trained (e.g., Ball et al., 2002; Willis et al., 2006; Klusmann et al., 2010; Bahar-Fuchs et al., 2017), while most studies demonstrated only a limited transfer to other cognitive functions and/or activities of daily living (Ball et al., 2002; Unverzagt et al., 2007).

In the past decades, several reviews have shown beneficial effects of cognitive interventions in healthy older adults (Papp et al., 2009; Martin et al., 2011; Tardif and Simard, 2011; Mowszowski et al., 2016; Mewborn et al., 2017; Webb et al., 2018). Each concluded that cognitive training can effectively improve various aspects of objective cognitive functioning, such as memory performance, executive functioning, processing speed, attention, fluid intelligence, and subjective cognitive performance. In a recent meta-analysis, authors Marusic et al. (2018b) reported the generalization of cognition-based interventions to a distal untrained domain, such as gait performance. The influence of cognition on mobility control in older adults has been shown previously (Heuninckx et al., 2005). This knowledge has opened new perspectives for cognitive training interventions for older population in general or those older individuals who are reluctant or not able to follow a physical activity intervention.

Recently, there is an increased use of CCT, which allows structured practice on standardized, and cognitively challenging tasks. CCT has several advantages over traditional drill and practice methods, including visually appealing interfaces, efficient and scalable delivery, the ability to measure performance and response time changes in multiple methods, and the ability to constantly adapt training content and difficulty to individual performance. The advantage of performing CCT in a supine/horizontal position opens new perspectives for implementing such a protocol in hospital/rehabilitation institutions. For a summary of non-physical approaches, readers are referred to Marusic and Grosprêtre (2018). The next section summarizes the impact of CCT during prolonged bed rest as a novel tool for mitigating negative effects of hospitalized older patients in acute phase after injury/surgery and/or in the subsequent rehabilitation process.

## Outcomes of the CCT Effects During Bed Rest

In this section we review the current evidence related to potential cognitive countermeasures in relation to extreme environments (e.g., experimental bed rest or hospitalization), which has not received much attention. A recent pilot study (Marusic et al., 2018a) showed the effectiveness of CCT intervention during bed rest in older adults. Sixteen healthy older male individuals (mean age of 60 years) were randomly assigned to an intervention and an active control group. Results revealed that CCT using virtual spatial navigation was effective and exerted long-lasting effects (up to 1.5 years after the study) as evaluated by improved performance on the virtual navigation task which was specifically targeted (Marusic et al., 2018a). In the same study, there were significant transfer effects of CCT, specifically on executive functions, attention, and processing speed (Marusic et al., 2018a). It was also observed that there was a detectable decline in the ability to perform complex motor-cognitive dual-tasks in the control group of older adults, but that CCT reduced the negative impact of bed rest on these integrated tasks, indicating better outcomes for the cognitively active intervention group (Marusic et al., 2015). Consequently, participants who followed the CCT protocol started their 28-day rehabilitation period (immediately after 14-day bed rest) from a higher functional and cognitive level.

Additionally, these data also revealed that CCT-related effects were also observed on the peripheral vascular function, perfusion assessments (Goswami et al., 2015b) as well as in the level of plasma brain-derived neurotrophic factor (BDNF) (Passaro et al., 2017). Older adults who underwent CCT did not show bed rest stress-related physiological changes (e.g., minimal changes in vascular function and increased level of BDNF) which indicate a preventive role of CCT during physiological deconditioning induced by 2-week bed rest immobilization. The mechanism of augmented BDNF levels after bed rest was attributed to a protective overshooting of the brain to counteract the bed rest-related negative effects (Soavi et al., 2016).

In addition to the above-mentioned findings, the electroencephalographic (EEG) recordings were used to evaluate the effects of 14-days bed rest on the brain neuroelectric activity. EEG results obtained with baseline eye-closed recordings showed that older adults who underwent CCT showed decreased power in lower delta and theta bands while control subjects showed significantly increased power in the same EEG spectral bands (Marusic et al., 2014a,b; Marušič, 2015). A so-called global “slowing” of the baseline, intrinsic EEG [e.g., increases in power in the slower delta range (2–4 Hz)], occurs with aging (Rossini et al., 2007). Vecchio et al. (2013) observed “slowing” of EEG in healthy older individuals progressing to mild cognitive impairment and probable Alzheimer’s disease. We concluded that increased spectral power in baseline EEG in control subjects but not in intervention subjects supported the notion that the CCT prevented negative effects of 14 days bed rest on brain baseline neuroelectric activity (i.e., increased power in EEG lower spectral bands) indicative of brain aging. Moreover, when analyzing event-related potentials (early perceptual processing of a stimuli), additional neuronal recruitment for the same amount of processing was observed only in the control group while participants in CCT group did not show the same trend (Marušič, 2015). In the same study, greater working memory enhancements (reduced P200 latency component) were observed in the CCT group, as compared to the controls.

In addition to CCT, other non-physical/cognition-based interventions, such as action observation and motor imagery might also be incorporated into experimental bed rest research. Motor imagery represents the mental simulation of an action without any corresponding motor output (Decety, 1996), while action observation (observing someone else’s movement) is known to activate the brain mirror neurons (Nedelko et al., 2010). The combination of both techniques induces even greater activity in motor areas of the brain as compared to either intervention alone (Taube et al., 2015). To date, no study has tested these non-physical techniques during a bed rest, which might open new perspectives for mitigating bed rest-related adaptation of the central nervous system (Van Ombergen et al., 2017b; Marusic and Grosprêtre, 2018).

## Conclusion and Future Directions

Overall, CCT interventions, developed from an underlying brain-based model (Marusic et al., 2018a), show that cognitive engagement during bed rest can trigger changes not only at the behavioral level, but also at the peripheral physiological (peripheral perfusion and blood BDNF level) and neuroelectric level. Thus, CCT intervention might represent a new promising approach for mitigating possible bed rest-associated physiological, functional, and cognitive declines, especially when motor execution is constrained or limited (e.g., during acute hospitalization) and may particularly be of special value for addressing the impact of bed confinement in older persons. Finally, it may also represent a promising research avenue as well as an option for a practical implementation in hospital settings and fall prevention programs.

## Author Contributions

UM drafted and wrote the article. VK performed to article drafting and final corrections. RP did final corrections. NG contributed to the article idea, drafting, and final corrections.

## Conflict of Interest Statement

The authors declare that the research was conducted in the absence of any commercial or financial relationships that could be construed as a potential conflict of interest.
